# Theory and Simulation Framework for the Relaxation of Nuclear Spin Order in Porous Media

**DOI:** 10.1021/acs.jpcb.2c03575

**Published:** 2022-08-17

**Authors:** Topaz A. A. Cartlidge, Thomas B. R. Robertson, Marcel Utz, Giuseppe Pileio

**Affiliations:** School of Chemistry, University of Southampton Southampton SO17 1BJ, U.K.

## Abstract

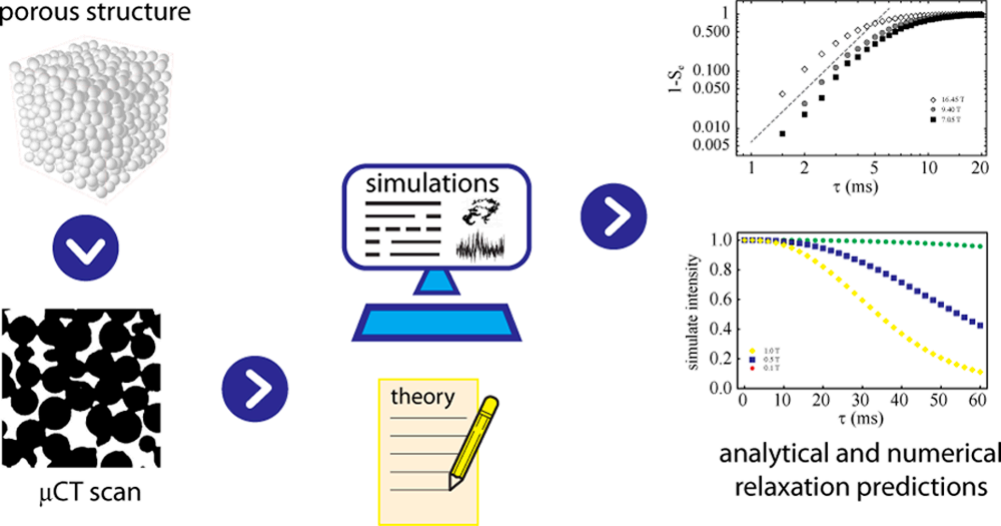

The theory of nuclear spin relaxation in a liquid permeating a solid structure of irregular geometry is examined. The effects of restricted diffusion and the demagnetizing field generated by an inhomogeneous distribution of magnetic susceptibility in the system are explored. A framework comprising Brownian Dynamics, average Hamiltonian theory, and Liouville-space spin dynamics is proposed for simulating nuclear spin relaxation in 3D models of random structures obtained from CT scans of actual samples. Simulations results are compared with experimental data. An analytical solution valid within approximation is also reported.

## Introduction

Nuclear spins diffusing in solution experience a variety of relaxation mechanisms that limit the lifetime of nonequilibrium spin order.^1^ Even the most fundamental interaction with a magnetic field can induce spin relaxation, when the field is inhomogeneous and the spins are free to diffuse in solution.^2,3^ At the basis of this effect, there is a phenomenon well-known to scientists performing diffusion NMR or MRI experiments, i.e., diffusive attenuation. Molecular translational diffusion in a spatially inhomogeneous magnetic field leads to a phase shift of the transverse spin order.^4^ This effect has been long exploited to measure diffusion coefficients and diffusion tensors.^5^ Diffusive attenuation also limits resolution in magnetic resonance imaging and, more relevant to this work, can lead to an additional source of relaxation for transverse spin order. The situation is exacerbated when molecular diffusion takes place in the voids of porous media. The difference in magnetic susceptibility between the solid framework and the voids in such systems give rise to local inhomogeneties in the magnetic field. Once a porous medium is placed in a static magnetic field, and the molecules move within and across pores, the nuclear spins carried by such molecules experience a magnetic field that fluctuates in both magnitude and direction. The effect of a randomly fluctuating magnetic field on nuclear magnetization has first been described by Redfield^6^ and Abragam;^1^ Callaghan^4^ provides a thorough review of the spin dynamics of molecules undergoing diffusion in the presence of magnetic field gradients.

For a spin diffusing in a porous system, the statistical properties of this random field depend both on the dynamics of diffusion and on the spatial inhomogeneity of the magnetic field, which are both influenced in a complex manner by the spatial structure of the pores. Quantitative prediction of the relaxation caused by this effect poses therefore several challenges:

1. measurement of the random spatial structure of the pores and representation by an appropriate statistical model;
2. calculation of the resulting inhomogeneous magnetic fields in the pore space;
3. modeling of the restricted diffusion^2^ of molecules in the pores and computation of the fluctuating field as a function of time;
4. conversion of the fluctuating field’s statistical properties into a quantum mechanical propagator that describes spin relaxation.

Various aspects of the effect of susceptibility inhomogeneities on NMR in porous media have been discussed in the literature,^7−15^ with most studies driven by the importance of diffusion NMR and MRI in materials science, in the petroleum industry, or in medicine.

The phenomenon has become particularly relevant for us in relation to our interests in long-lived spin order.^16^ In recent studies, we have exploited the long position-encoding time offered by long-lived spin order in diffusion-NMR applications to localize spins over long times or long distances in magnetic resonance imaging,^17^ measure very slow flow,^18^ extend the scope of diffusive-diffraction *q*-space imaging,^19^ or measure tortuosity in porous media.^20^ The long-lived spin order is created from single quantum coherences, which are converted by special pulse sequences. While the long-lived order is unaffected by diffusive attenuation, this does not apply to the single quantum coherences it originates from. Creation and observation of long-lived spin order is severely limited in porous media at high magnetic fields. The pulse sequences, for example M2S/S2M^21^ or SLIC,^22^ must be synchronized to the actual spin system’s parameters and therefore cannot be kept conveniently short to minimize the effects of diffusive attenuation. In particular, the M2S/S2M sequence relies on relatively long and repeated echoes, while SLIC is based on a long spin lock. In either case, coherence decay typically results in a less efficient interconversion between long-lived spin order and single quantum coherence. As a result, when these pulse sequences are run in certain porous media no conversion of transverse into long-lived spin order is obtained at all. This is somewhat bizarre considering the fact that the long-lived spin order itself is unaffected by field gradients in the sample (unless the field varies on a molecular length scale). Motivated by the necessity to characterize this phenomenon, we have developed a simulation framework that predicts the relaxation of spin states due to susceptibility inhomogeneities in a porous system of arbitrary complexity. It relies on microcomputed X-ray tomography scans (μCT) of the porous structure, from which the position-dependent field is computed by a discrete Fourier transform approach. The same structure is used for a Brownian dynamics simulation of diffusion in the liquid phase. The resulting trajectories then serve as input to an average Hamiltonian approach to compute the relaxation propagator. As we discuss in the following, diffusive attenuation can also be treated theoretically, but fairly strong assumptions on the stochastic independence of diffusion and the random magnetic fields must be made.

We tested the predictions from both simulation and theory against experimental data taken at three different magnetic fields on three model porous media made up by randomly packed polyethylene spheres of different size imbibed with a tetramethylsilane solution in methanol-*d*_4_.

## Theory and Simulation Methodology

### Magnetic Fields in Porous Media

The magnetic field at each point in the structure of a porous medium can be thought of as the superposition of the magnetic field generated by the dipole magnetic moments present at each point in the structure. In general, the magnetic induction **B** (which governs the quantum dynamics of nuclear spins) is related to the magnetic field **H** and the magnetization **M** as
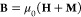
1In diamagnetic and paramagnetic materials, the magnetization is proportional to the magnetic field **H**_0_ as in
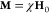
2In the present context, χ is a piecewise-constant function of position. If we assume a porous solid described by the structure function *S*(**r**), the function χ(**r**) is given by
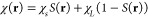
3where χ_*s*_ and χ_*L*_ are the volume susceptibilities of the solid and liquid phases, respectively. Obviously, this continuum treatment is only applicable for structures that are much larger than atomic sizes. This is adequate in the present context, as we are dealing with micrometer-sized pores. Without loss of generality, we assign the *z*-axis to be aligned with the external magnetic field, such that **H**_0_ = *H*_0_**e**_*z*_. The magnetization then becomes
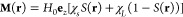
4The magnetic field has to satisfy Maxwell’s equations
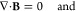
5
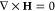
6By inserting (1) into (5), we obtain
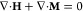
7The magnetic field can be separated into a constant (external) field, which we assume to be aligned with the *z*-axis, and a position dependent field **H**_*d*_(**r**) as in
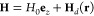
8For historical reasons, **H**_*d*_ is known as the ”demagnetizing field”. In the presence of piecewise-constant magnetic susceptibility, **H**_*d*_ varies continuously as a function of position. It is these variations that cause diffusive attenuation. Introducing (8) into (7), we obtain
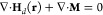
9With (4), and noting that ∇·= (∂_*x*_, ∂_*y*_,∂_*z*_)·, this becomes
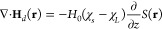
10Since the structure function *S*(**r**) is constant everywhere except on its boundaries, this differential equation, together with Ampere’s law (6), represents an inhomogeneous boundary value problem, which can be solved using the Green’s function
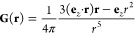
11The demagnetizing field is then given by the convolution^23,24^
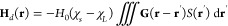
12Note that μ**G**(**r** – **r**′) represents the magnetic field generated at position **r** by a point magnetic dipole μ**e**_*z*_ located at **r**′. The demagnetizing field is therefore the result of local magnetic dipoles that are induced in the paramagnetic or diamagnetic material by the external field **H**_0_.

It will prove convenient to formulate the problem in dimensionless form. In this way, the results obtained can be scaled to different pore sizes, diffusion coefficients, and magnetic susceptibility differences. We therefore introduce a dimensionless position vector
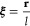
13where *l* is a length scale which can be chosen freely. It could, for example, represent the average pore size, or the voxel size of the CT data. The Green’s function **G**(**r**) is homogeneous of degree −3; i.e., it has the property
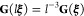
14The convolution (12) can therefore be rewritten as
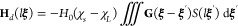
15This means that the demagnetizing field is *length-scale invariant*. Using the Fourier convolution theorem, the demagnetizing field can be computed as
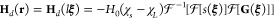
16where  is the three-dimensional Fourier transform,  is its inverse, and *s*(***ξ***) = *S*(*l**ξ***) is the dimensionless (or scale-free) structure function. This formulation is convenient for numerical computations, since  need only be computed once, and results derived for one structure function *s*(***ξ***) are valid at any length scale.

### Fluctuating Field Experienced by Diffusing Spins

As the molecules diffuse in the pore structure, they randomly sample the demagnetizing field, such that each diffusing molecule experiences a field that fluctuates randomly as a function of time. The statistical properties of these fluctuations arise as a combination of the statistics of the Brownian motion of the molecules and those of the demagnetizing field. These two random processes are potentially highly correlated—after all, the structure *S*(**r**) both constrains the diffusion *and* gives rise to the demagnetizing field.

The Brownian motion of a diffusing molecule makes its position a random function of time **r**(*t*). If the diffusion is free, then this function represents a Wiener process with
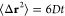
17where Δ**r** = **r**(*t*) – **r**(0), and *D* is the free diffusion coefficient. This continues to be valid for the restricted diffusion inside the pore structure *S*(**r**) for diffusion times that are short compared to *a*^2^/6*D*, where *a* is a measure of the pore size. For longer times, collisions with the pore walls cause deviations from free diffusion, and Δ**r**(*t*) is no longer a Wiener process. The motion of the particle inside the pore structure makes the magnetic induction experienced by the particle a stochastic function of time:
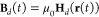
18

### Averaged Propagator

As a model system, we consider an ensemble of molecules containing a single nuclear spin with spin quantum number ^1^/_2_. In addition to the fluctuating demagnetizing field **B**_*d*_(*t*), the molecules are exposed to an external magnetic field assumed along the *z*-direction of the laboratory frame, *H*_0_**e**_*z*_. Since the transverse components of the demagnetizing field give rise to terms in the spin Hamiltonian that do not commute with the Zeeman term arising from *H*_0_**e**_*z*_, an average Hamiltonian approach is required.^25^

#### Average Hamiltonian

The total time-dependent Hamiltonian *Ĥ*(*t*) for a spin-^1^/_2_ system diffusing through a demagnetizing field and in the presence of a large external field *H*_0_**e**_*z*_ is given by
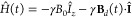
19where^26^
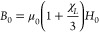
20As mentioned above, the two terms in eq 19 do not commute with each other. However, in many cases of interest, the fluctuations of **B**_*d*_ are on a much slower time scale than the period of the Larmor precession. Formally, this can be expressed by the condition
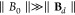
21In this case, it makes sense to work with the average Hamiltonian over a full Larmor precession. To do so, we transform the Hamiltonian into the interaction frame of the Zeeman term of the Hamiltonian, *Ĥ*_*z*_, as in
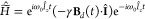
22where ω_0_ = −*γ B*_0_ is the Larmor frequency. Equation 22 can be expanded by explicitly solving the frame rotation to obtain
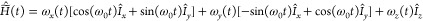
23with ω_*x*_(*t*) = −*γB*_*d*,*x*_(*t*), ω_*y*_(*t*) = −*γB*_*d*,*y*_(*t*), and ω_*z*_(*t*) = −*γB*_*d*,*z*_(*t*). Assuming the time dependence of the fluctuating field to be slow compared to the Larmor period, the time-dependent Hamiltonian above can be averaged over a Larmor period to give rise to the Magnus series:^27^
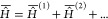
24where the terms on the right-hand-side are the first, second, third, and so on orders of the expansion. The first two orders are given by^25^
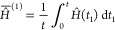
25
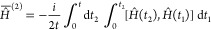
26And, for the current case we obtain
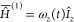
27
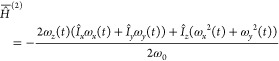
28In the context of this paper, only the first-order averaged Hamiltonian will be considered. This is because in diamagnetic and paramagnetic systems |χ_*s*_ – χ_*L*_| ≪ 1. Since ω_*x*_, ω_*y*_, and ω_*z*_ scale linearly with the susceptibility difference χ_*s*_ – χ_*L*_, the second order term is proportional to (χ_*s*_ – χ_*L*_)^2^ and can therefore be neglected.

#### Single Spin–Echo Propagator

**Figure 1:**
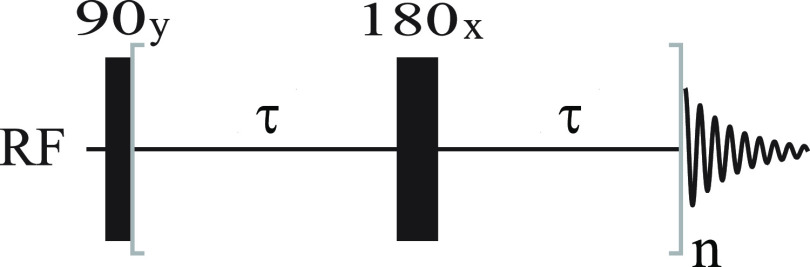
Spin echo pulse sequence used for single echo experiments (*n* = 1 and variable τ) or measurements of *T*_2_ (τ fixed as specified and variable *n*).

The central task of our simulation framework is to numerically compute the averaged propagator for a spin-^1^/_2_ system, subjected to the first-order truncated averaged Hamiltonian in eq 27, during a single spin–echo pulse sequence consisting of a π_*x*_ pulse separated by equal time delays τ (see Figure 1). The propagator for such a sequence is given by
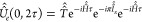
29where *Ĥ̂* is the commutator superoperator of the Hamiltonian and *T̂̂* is the Dyson time-ordering superoperator. The first-order truncated average Hamiltonian *Ĥ̂*^(1)^(*t*) commutes with itself at all times. Introducing the following notation for rotation superoperators:
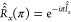
30the propagator in eq 29 can be expanded to first order as
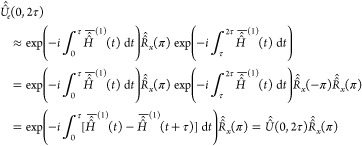
31where *Û̂*(0, 2τ) is the part of the echo propagator associated with the decay of transverse magnetization during the time interval 2τ. Using the explicit form of the average Hamiltonian in eq 27 and recalling the definition ω_*z*_(*t*) = −*γB*_*d*,*z*_(**r**(*t*)), we obtain
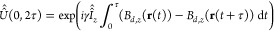
32with *B*_*d*,*z*_ = μ_0_*H*_*d*,*z*_ as calculated in eq 16. It can already be appreciated that the propagator *Û̂*(0, 2τ) will approach unity in the limit of echo times that are short compared to the correlation time of the fluctuating Larmor frequency ω_*z*_(*t*). The effects of the random field experienced by the diffusing spins can therefore be arbitrarily scaled down by applying π pulses separated by short enough time intervals. In practice, however, the available (and tolerable) RF power impose limits to how fast these π pulses can be repeated. Moreover, in several situations, such as in the case of pulse sequences that prepare singlet spin order, the echo time cannot be chosen arbitrarily short but it is rather dictated by the spin system parameters.

In an ensemble of molecules containing nuclear spins in a fluid phase permeating a porous solid, ω_*z*_(*t*) becomes a random variable, due to the diffusion of the particle within the pores. Hence, in order to describe the global evolution of the system, we need the ensemble average of eq 32, formally given by
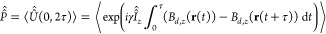
33

### Relaxation of Longitudinal and Transverse Spin Order

The averaged propagator in eq 33 can be used to study the dynamics of the density operator during the pulse sequence in Figure 1. Still in the case of a single spin-^1^/_2_ system, assuming thermal equilibrium at the beginning of the experiment, the density operator immediately after the first 90 deg pulse is
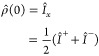
34containing the transverse spin order, i.e., single quantum coherences. If we adopt the following basis set for the Liouville space: {*Î*^–^, *Î*_*z*_, 1̂, *Î*^+^}, then the density operator at time *t* = 0 is represented by the following column vector:
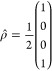
35The density operator at time 2τ is then calculated from
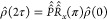
36In the same basis, the propagator *Û̂*_*e*_(0, 2τ) is represented by the matrix
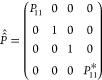
37with
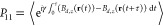
38and, *R̂̂*_*x*_(π) is represented by
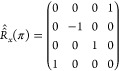
39Hence, we find
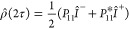
40Giving the fact that in quadrature detection we only observe the −1Q coherence we can restrict our attention to the term
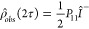
41The ensemble averaged exponential coefficient describes the decay of the transverse magnetization as the spins diffuse in the inhomogeneous magnetic field. The factor can be numerically calculated as explained below. Incidentally, it is easy to see that this phenomenon applies to transverse but not to longitudinal spin order, and therefore, it contributes to *T*_2_ but not to *T*_1_ relaxation. In the basis adopted, the longitudinal spin order is represented by the column vector
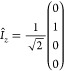
42which is clearly left unaltered by the averaged propagator in eq 37 and simply switched in sign by the propagator in eq 39. Indeed, a signature of the presence of susceptibility inhomogeneities induced relaxation is the experimental observation that *T*_1_ remains unaffected, unlike *T*_2_. Note that this fact is not dependent on the length scale of the susceptibility inhomogeneities, but follows purely from the average Hamiltonian (27) and (28).^2^

### Analytical Solution

Let us consider the propagator element *P*_11_ in eq 38, which governs the evolution of observable transverse spin order. For small enough diffusion displacements one can use a first-order Taylor expansion. It is convenient to choose the middle of the spin echo interval (the time of the inversion pulse) as the expansion point and the time origin *t* = 0. This gives
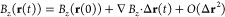
43where ∇*B*_*z*_ is the field gradient experienced by the diffusing spin at time *t* = 0. This leads to
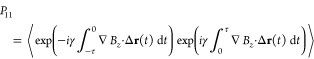
44
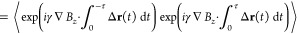
45Since the diffusion displacements Δ**r** before and after *t* = 0 are statistically independent, this may be simplified to
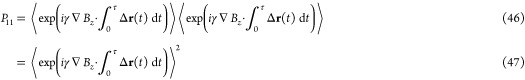
The integral in eq 47 is the average diffusion displacement:
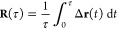
48With this substitution, we obtain
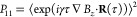
49To compute the ensemble average, we write the joint distribution function of the magnetic field gradient and the average diffusion displacement as
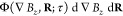
50The ensemble average of any quantity *A* can then be expressed as the integral
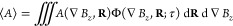
51As outlined before, the distribution function Φ(∇*B*_*z*_, **R**; τ) is unknown, and in general, it contains complex information on the interplay between the fluctuating field and restricted diffusion. A numerical approach to sample the distribution function Φ is described below. An analytical solution requires some simplifying assumptions, which will have to be justified in view of experimental results. If we assume the average diffusion displacement and the magnetic field gradients to be uncorrelated, the distribution function factorizes into
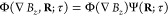
52

This is a rather drastic assumption, which is only valid if the diffusive attenuation is so efficient that relaxation is essentially complete on the time scale that is needed for the molecules to reach the walls of the pores. We would therefore expect this assumption to be valid at high magnetic fields, where the magnetic field variations are large, and for large pore sizes. **R** is given by the integral (48). If we further assume that over short enough times τ the diffusion remains unrestricted by the pore walls, Δ**r** is a three-dimensional Wiener process, with components
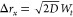
53where *W*_*t*_ is the standard Wiener process with variance σ^2^(*W*_*t*_) = *t*, and likewise for the other two components. The integral (48) can therefore be evaluated as
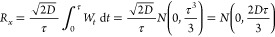
54where *N*(0,1) represents a Gaussian random process with zero mean and unity variance. We therefore have
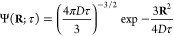
55The matrix element *P*_11_ can therefore be expressed as
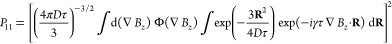
56The spatial integral is in fact the three-dimensional Fourier transform of a Gaussian function, and can be evaluated analytically. This leads to
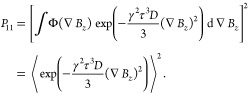
57This expression can be used to compute the propagator if the field gradient ∇*B*_*z*_ is known. In the special case of ∇*B*_*z*_ = *G* = const., this reduces to
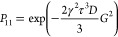
58which is the well-known echo attenuation coefficient derived by Carr and Purcell for a particle diffusing in a constant magnetic field gradient.^4,28^

The ensemble average can be evaluated numerically as a volume average over the liquid phase. Using our earlier definition of the structure function *S*(**r**), which is unity in the solid and vanishes in the liquid, we obtain
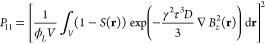
59where *ϕ*_*L*_ is the volume fraction of the liquid. The exponential in eq 57 can be expanded in a Taylor series. To leading order, this yields
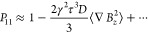
60The field gradient scales proportionally to the susceptibility difference. Since the demagnetizing field is independent of length scale for a given spatial structure, the field gradient is inversely proportional to the pore size *a*. We can therefore set ⟨∇*B*_*z*_^2^⟩ ≈ *B*_0_^2^(χ_*L*_ – χ_*s*_)^2^/*a*^2^, and arrive at the scaling law
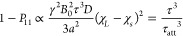
61which remains valid under the assumptions discussed above, as long as the right-hand side is much smaller than unity. The characteristic attenuation time is given by
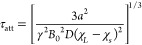
62

## Numerical Simulation Framework

**Figure 2:**
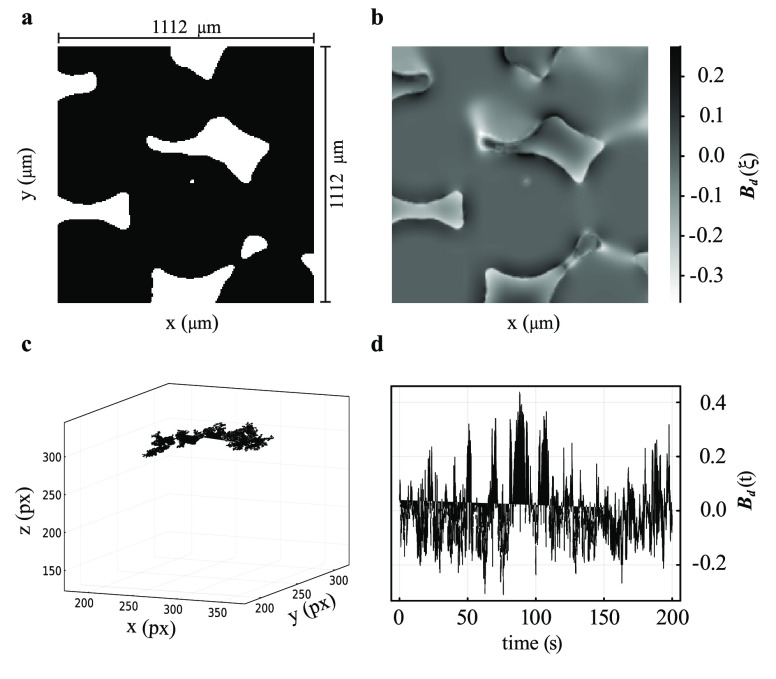
Typical results of a numerical simulation on a sample of packed PE beads showing (a) a 200 × 200 expansion of the binarized μCT image (1 px = 5.56 μm), (b) the dimensionless demagnetization field **B**_*d*_(**ξ**) calculated over the same region as in a, (c) the positions covered by a single molecule during a random walk through the pores of the structure, and (d) the demagnetizing field experienced by the molecule as a function of time, **B**_*d*_(*t*). Simulation parameters are collected in Table 1.

To numerically evaluate the propagator element *P*_11_ in eq 38, we have set up a simulation framework (available in Mathematica^29^ and Julia^30^ languages) which consists of the following steps:

1. *Digital rendering of the porous structure.* A microcomputed tomography 3D image of the porous medium is acquired and binarized to obtain the structure function, *s*(**ξ**). This function contains information about the structural nature of the porous structure at each position **ξ**, namely *s*(**ξ**) = 1 if **ξ** falls within the solid matrix and *s*(**ξ**) = 0 if **ξ** falls within a pore. Note that such knowledge of the structure is discrete and has the same resolution of the CT scan (see Figure 2a) . The digitized CT structure is then Fourier transformed to yield  which is required in eq 16 to calculate the field **H**_*d*_(**r**).
2. *Compute the magnetic field within the porous medium.* The demagnetizing field **H**_*d*_(**r**) is computed on a 512^3^ mesh of the CT image (taken at the center of the original image) using a discrete version of eq 16. The demagnetizing field is then linearly interpolated to create a finer resolution.
3. *Simulate Brownian diffusion.* Using Brownian dynamics, the discrete translational trajectory resulting from the molecular random walk within and across the pores of the structure is calculated as follows:
  (a) An initial point **ξ**_0_ = {*x*_0_, *y*_0_, *z*_0_} is randomly chosen to fall within a pore in the structure, i.e., *s*(**ξ**_0_) = 0 and a step counter *j* is set to 1.
  (b) A random step is attempted by setting **ξ**_*j*_ = {*x*_*j*–1_ + *δx*_*j*_, *y*_*j*–1_ + *δy*_*j*_, *z*_*j*–1_ + *δz*_*j*_}, with the step increments *δx*_*j*_, *δy*_*j*_ and*δz*_*j*_ chosen randomly from a Gaussian distribution of zero mean and standard deviation , where *D* indicates the isotropic self-diffusion coefficient of the molecule carrying the spin and *t*_*s*_ is the time increment.
  (c) The point **ξ**_*j*_ is checked such that if *s*(**ξ**_*j*_) = 0, then the new point is still in the pore space, the position **ξ**_*j*_ is stored in the trajectory array **p** = {**ξ**_*j*_}, and *j* is incremented by 1. If *s*(**ξ**_*j*_) = 1, a new random step is attempted by returning to step b.
  (d) Steps b and c are repeated *N* times. This produces a discrete trajectory of *N*_*s*_ entries (in general, *N*_*s*_ ≤ *N*, due to the rejection of steps colliding with the solid phase). To produce a statistically accurate description of spins randomly diffusing in porous media, the procedure above must be repeated for a (large) number of initial positions so as to simulate the different translational trajectories accessible to the different molecules. The total time for each trajectory *N*_*s*_*t*_*s*_ must be on the order of the experimental time of interest, typically in the range between tens and hundreds of milliseconds in a liquid-state NMR experiment. In this paper, we have adopted the alternative approach consisting of simulating a very long trajectory (hundreds of seconds) for a single molecule and dividing this into many shorter subtrajectories. The two approaches produce statistically equivalent results if the pore structure is sufficiently interconnected as we believe it is the case of our test samples below. The result of a typical random walk simulation performed on the test samples described below is shown in Figure 2c.
4. *Compute fluctuating field experienced by diffusing spins.* Once the field map is generated (step 2) and molecular positions as a function of the time are computed (step 3), the fluctuating magnetic field experienced by the spins along their translational trajectory becomes available. The field experienced by the spin at the *j*th step of its random diffusion through the porous medium is
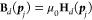
63Since each point in the trajectory is spaced in time by *t*_*s*_, the field experienced by the spin at the time *t* = *n*_*s*_*t*_*s*_ (i.e., after *n*_*s*_ steps) is given by
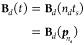
64The function **B**_*d*_(*t*) is a fluctuating function and is required in the calculation of the propagator element *P*_11_ in eq 38. The fluctuating field experienced by molecules during their walk in a simulation using the test samples described below is shown in Figure 2d.
5. *Compute the averaged propagator element.* The propagator in eq 38 is averaged over a large number of individual trajectories to accurately reflect the dynamics in a macroscopic ensemble of spins. To do so we have divided the whole trajectory made by *N*_*s*_ points and total duration *N*_*s*_*t*_*s*_ in a number of subtrajectories, *N*_*st*_ = *N*_*s*_/*n*_*p*_, each containing the number of points *n*_*p*_ = τ/*t*_*s*_. In a typical calculation, *N*_*s*_ is set to 10 million points and *t*_*s*_ to 20 μs; thus *n*_*p*_ = 50 and *N*_*st*_ = 2 × 10^5^.

The required propagator element is then calculated as
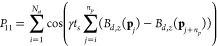
65where only the real part of the complex exponential function appearing in eq 38 needs to be calculated since the imaginary part vanishes due to the detailed balance.

All parameters used in the simulations are collected in Table 1.

**Table 1:** Simulation Parameters

parameter	symbol	value
CT image resolution	*l*	5.56 μm^a^
		9.31 μm^b^
diffusion coefficient TMS in MeOD	*D*	2.4 × 10^–9^ m^2^ s^–1^
magnetic susceptibility MeOD	χ_*Me*_	–6.96 ppm^c^
magnetic susceptibility PE	χ_*PE*_	–9.67 ppm^d^
susceptibility difference	Δ*χ* = χ_*Me*_ – χ_*PE*_	2.71 ppm
number of steps in the walk	*N*_*s*_	10^7^
step duration	*t*_*s*_	20 μs
total walk duration	*T*_*s*_ = *N*_s_*t_*s*_	200 s
average step distance	*d*_*s*_ = √(2*Dt*_*s*_)	0.253 μm
average total distance	*D*_*s*_ = √(2*DT*_*s*_)	800 μm

a Sample PES.

b Samples PEM and PEL.

c Reference (31).

d Reference (32).

## Experimental Materials and Methods

In order to compare numerical predictions with experiments so to validate our simulation framework we have measured the signal area as a function of the echo time τ using the pulse sequence in Figure 1 with *n* = 1 and in a number of model porous media made up by polyethylene beads of different size. These measurements have been done at three different values of the static magnetic field. For completeness, we also measured *T*_1_ and *T*_2_ decay times in all samples described below.

### Samples

The model porous media samples used in this work were made by random packs of polyethylene beads. All samples were prepared in a 5 mm NMR tube sourced from Wilmad LabGlass. The total packing height was 5 cm in order to fully encompass the probe coil region with sufficient excess to ensure that the packing was as uniform as possible across the region of interest. The packing was done by weighing out ca. 1.2 g (bead size dependent) of the polyethylene beads and adding this to the NMR tube in between 2 and 4 aliquots, depending on the bead size. Between each aliquot gentle manual tapping was undertaken to aid in packing. Polyethylene microspheres were purchased from Cospheric’s CMPS products in sizes 212–250, 500–600, 710–850, and 1000–1180 μm and were used without further purification. The bead packings were imbibed with a 0.193 M stock solution of tetramethylsilane (TMS) in deuterated methanol (MeOD). Both solvent and solute were purchased from Sigma-Aldrich and used without further purification. Where required, gentle manual shaking was utilized to remove any visible air bubbles. Table 2 summarizes the sample composition and nomenclature.

**Table 2:** Sample Composition and Nomenclature

sample name	bead material	bead size/μm	imbibing solution/M
PES	polyethylene (PE)	212–250 (S)	0.19M TMS in MeOD
PEM		500–600 (M)	0.193M TMS in MeOD
PEL		1000–1180 (L)	0.193M TMS in MeOD
BLK	–	–	0.193M TMS in MeOD

### NMR

NMR data were collected at three different values of the static magnetic field, namely 7.05, 9.4, and 16.45 T. Data at 7.05 T was collected in a Bruker Avance III 300 MHz spectrometer running topspin 3.5 and equipped with a 10 mm MICWB40 Bruker probe. Data at 9.4 T was collected on a Bruker Avance Neo 400 MHz spectrometer running topspin 4.0.8 and equipped with a 10 mm BBO Bruker probe. Data at 16.45 T was collected on a Bruker Avance Neo 700 MHz spectrometer running topspin 4.0.7 and equipped with a Bruker CPP TCI 700S3 probe. For all data collected, a single shim file was saved based on shimming the beads-free BLK sample (see Table 2). The same sample was also used to optimize the 90 and 180 deg pulses at all fields. Data fitting and relaxation values were calculated using a custom-made palette running within the Wolfram Mathematica software. In the following, we will refer to *T*_1_, *T*_2_, and single echo experiments. *T*_1_ measurements use a standard saturation recovery pulse sequence. *T*_2_ experiments use a standard CPMG pulse sequence like the one reported in Figure 1 run with fixed τ and variable *n*. Single echo experiments are also based on the pulse sequence in Figure 1 but are run with *n* = 1 and at variable τ values.

### μCT

**Figure 3:**
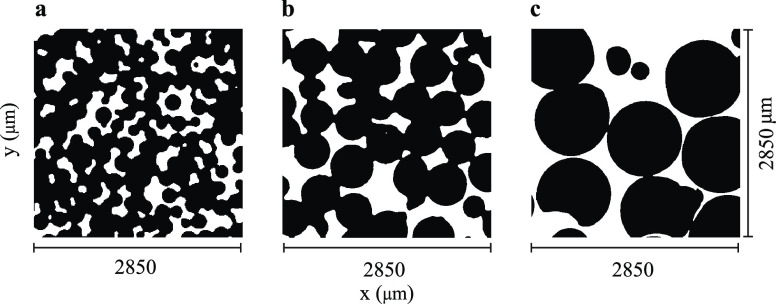
Central *xy*-plane slice of the μCT images taken on the three polyethylene beads packings studied in this work: (a) bead size 212–250 μm, resolution 9.31 μm/pixel; (b) bead size 500–600 μm, resolution 5.56 μm/pixel; and (c) bead size 1000–1180 μm, resolution 5.56 μm/pixel.

To obtain the digitized structure function *S*(**r**), we used microcomputed tomography (μCT). CT images of the three model porous media in Table 2 were collected using a modified 225 kVp Nikon/Xtek HMX scanner. To improve contrast, the sample packings were prepared as described above but in custom-made 10 mm poly(carbonate) tubes formed from a cut section of 10 mm outer diameter and 1.5 mm wall-thickness tubing (Clear Plastic Supplies, U.K.). For all samples, 3 g of microspheres was added to the tube in a number of small aliquots. At each addition, the sample was gently tapped to improve the packing. The packed beads were imbibed with a 0.193 M solution of TMS in MeOD and manually shaken to remove any visible air bubbles. The tube was sealed and left overnight before imaging. μCT images were processed using the ImageJ software. Raw data comprising the whole sample where cut to manageable sizes for simulations (512 × 512 × 512 pixels taken from the center of the reconstructed volume). These reduced-volume images were filtered and binarized. A slice of each data set is shown in Figure 3. Binarization was achieved using ImageJ’s automatic threshold tool with thresholds calculated for each slice and manually checked after binary conversion. A median filter of 3 pixels was applied to the volume before binarization in order to minimize noise and remove artifacts. A further median filter of 2 pixels was applied following binarization.

## Results and Discussion

### 1D NMR Spectra

**Figure 4:**
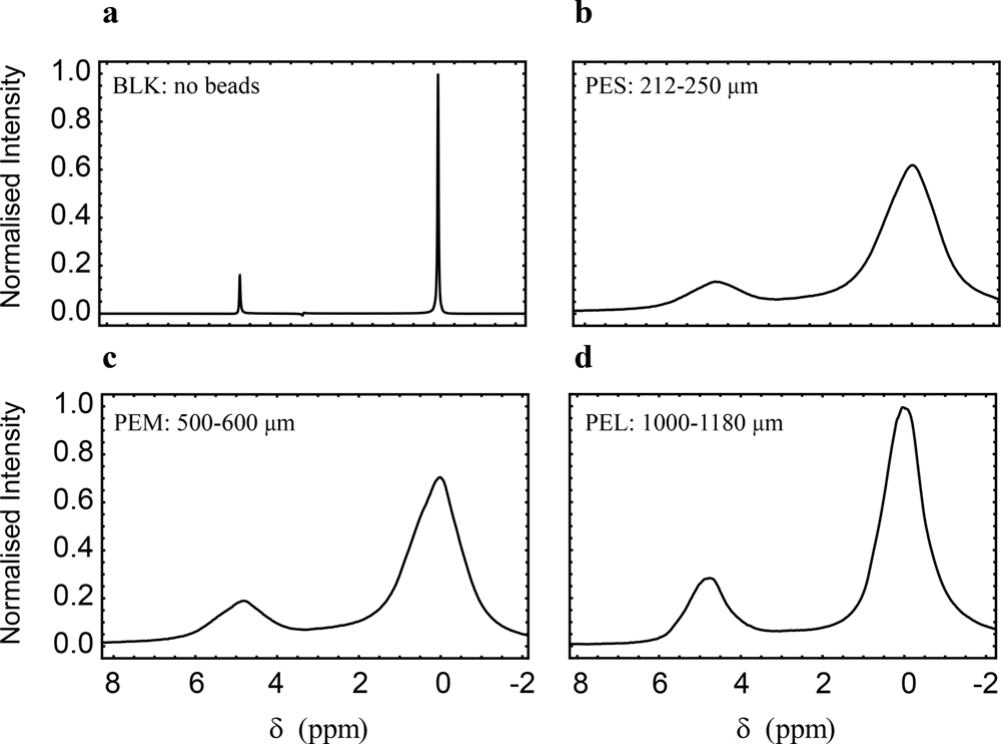
1D NMR spectra of sample (a) BLK, (b) PES, (c) PEM, and (d) PEL taken at 16.45 T (see Table 2). Spectra b, c, and d are normalized to the same intensity scale for comparison.

1D NMR spectra of the four samples in Table 2 have been recorded at 16.45 T to examine the effect of the susceptibility inhomogeneities on the spectral line width. The spectra are compared in Figure 4. The peak at 0 ppm is due to TMS while the peak at 4.8 ppm is due to the residual protonated methanol. As expected, the presence of the PE beads in the sample severely compromises the spectral resolution. The effect is more severe for the sample with small beads (PES) and lessens as the bead size increases. Clearly, the line width is dominated by the field inhomogeneity due to the susceptibility differences between the beads and the solvent. However, the magnetic field is scale-invariant, as discussed above. The fact that the line width does depend on the length scale, if only weakly, already suggests that there must be a contribution to the line width from a *T*_2_ relaxation mechanism that becomes more effective at smaller length scales.

### Relaxation Measurements

The *T*_1_ relaxation decay constants of all samples were measured, at each magnetic field, using a conventional saturation recovery pulse sequence with the relaxation variable delay time ranging from 0.1 ms to 32 s. The results of these experiments are reported in Table 3.

**Table 3:** *T*_1_ Relaxation Decay Constants for All Samples in This Work

	*T*_1_ (s)		
sample name	7.05 T	9.4 T	16.45 T
BLK	4.7 ± 0.1	5.4 ± 0.1	5.7 ± 0.1
PES	4.5 ± 0.1	5.0 ± 0.1	5.6 ± 0.1
PEM	4.8 ± 0.2	5.0 ± 0.1	5.7 ± 0.1
PEL	4.6 ± 0.1	5.1 ± 0.1	5.6 ± 0.1

As expected, the beads and resulting susceptibility inhomogeneities do not alter *T*_1_. Conversely, as established in the theory section above, the relaxation decay constant of transverse spin order, *T*_2_, measured using a train of spin echoes (Figure 1 with fixed τ and variable *n*—a.k.a. the Carr–Purcell–Meiboom–Gill method) must depend on the actual echo time chosen in the experiment. To highlight this effect, we have measured the *T*_2_ decay constants in the PEM sample, at the three different magnetic fields and for a series of echo times, as indicated in Table 4.

**Table 4:** *T*_2_ Relaxation Decay Constants for Sample PEM Using Different Echo Times

	*T*_2_ (s)		
τ (ms)	7.05 T	9.4 T	16.45 T
0.5	1.93 ± 0.02	1.84 ± 0.02	1.18 ± 0.02
1	1.30 ± 0.03	1.29 ± 0.03	0.61 ± 0.02
2	0.64 ± 0.03	0.60 ± 0.01	0.25 ± 0.01
4	0.29 ± 0.02	0.23 ± 0.01	0.09 ± 0.01
8	0.13 ± 0.01	0.09 ± 0.01	0.05 ± 0.01
16	0.10 ± 0.01	0.06 ± 0.01	0.04 ± 0.01

The data in Table 4 show how the measured *T*_2_ value gets shorter and shorter as the echo time is increased. This is because the spins can diffuse further away from their original positions during an increased echo time. The values of *T*_2_ also become consistently smaller as the static magnetic field is increased as the effect is directly proportional to the magnetic field (see eq 41).

### Diffusion Experiments

The value of the unrestricted diffusion coefficient of TMS in MeOD is required in the random walk part of the simulation framework. This coefficient has been experimentally measured at 7.05 T using sample BLK (see Table 2) and a conventional pulsed gradient stimulated echo (PGSTE) sequence that used bipolar gradients and one spoiler gradient. The measurement returned *D* = (2.37 ± 0.03) × 10^–9^ m^2^ s^–1^.

### Single-Echo Experiments

**Figure 5:**
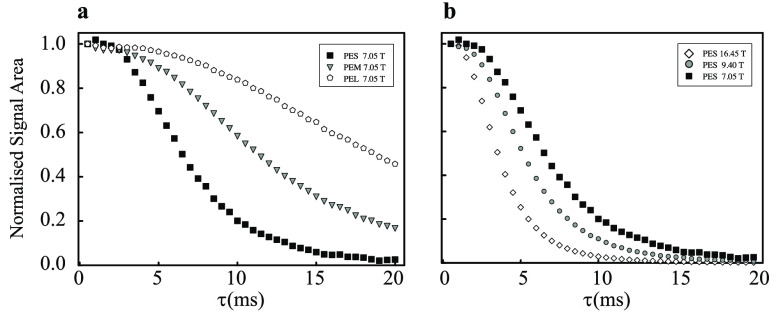
Experimental data from single-echo experiments for (a) samples PES (squares), PEM (triangles), and PEL (pentagons) at 7.05 T and (b) sample PES at 16.45 T (diamonds), 9.4 T (circles), and 7.05 T (squares) (see Table 2). The signal area has been plotted so that the shortest time point has intensity 1.

The results of single-echo experiments, using the pulse sequence shown in Figure 1 with *n* = 1, are compiled in Figure 5a for different magnetic fields and Figure 5b for different bead sizes. All decay curves exhibit a similar shape, reminiscent of a Gaussian function. At short echo times, the normalized signal area is constant, and starts to decay only once the echo time reaches several milliseconds. Clearly, smaller beads and larger magnetic fields lead to faster decay.

**Figure 6:**
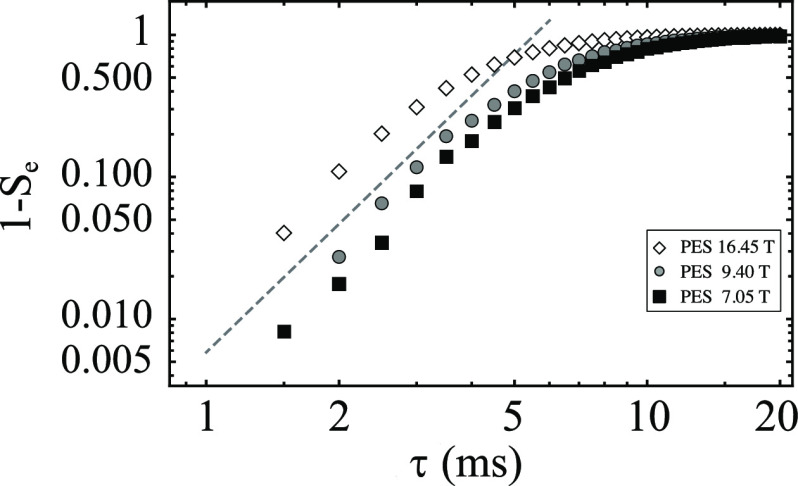
Log–log plot of experimental data from single-echo experiments (S_e_) for sample PES at 16.45 T (diamonds), 9.4 T (circles), and 7.05 T (squares). The dotted gray line is the plot of τ^3^ and is meant to guide the eyes to a slope of 3. Experimental data refers to the peaks’ signal area normalized so that the shortest time point has unitary area.

The scaling law, eq 61, predicts a decay with 1 – *P* ∼ τ^3^. Figure 6 shows a doubly logarithmic plot of the experimental results. In close agreement with the theoretical prediction, the data present as an initial straight line with a slope close to 3. At longer echo times, the decay slows down with *P* approximating zero.

**Figure 7:**
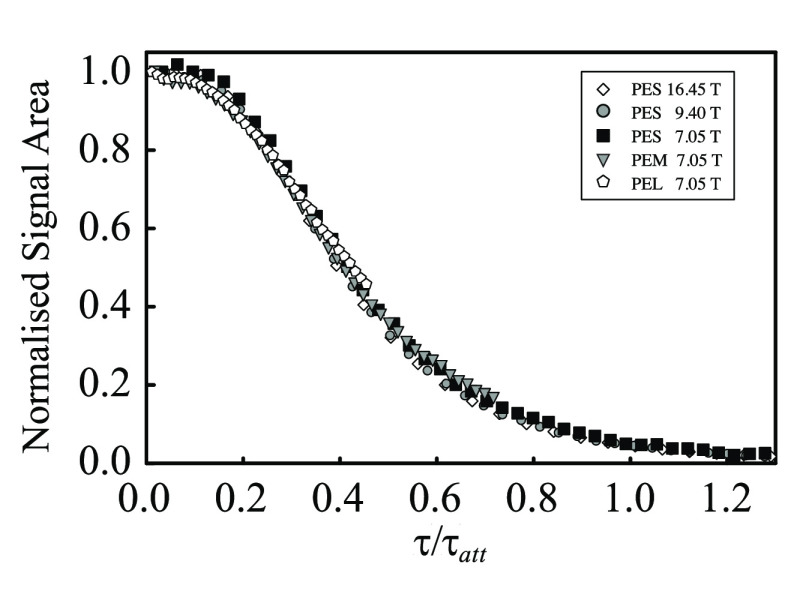
Experimental signal area from single-echo experiments for sample PES at 16.45 T (diamonds), 9.4 T (circles), and 7.05 T (squares) and samples PEM (triangles) and PEL (pentagons) at 7.05 T (see Table 2). The signal area has been normalized so that the shortest time point has unitary area.

Agreement with the theory developed above is further corroborated by plotting all available data as a function of the reduced echo time . The characteristic attenuation time has been calculated according to eq 62, with the bead diameter used for the characteristic length scale *a*. This is shown in Figure 7. All data points, taken with different bead sizes and different magnetic fields, collapse onto a single master curve. This behavior is directly predicted by eq 57. The experimental data therefore directly validate the assumptions made in developing the theory, first and foremost the stochastic independence of the diffusion process and the irregular magnetic field distribution.

**Figure 8:**
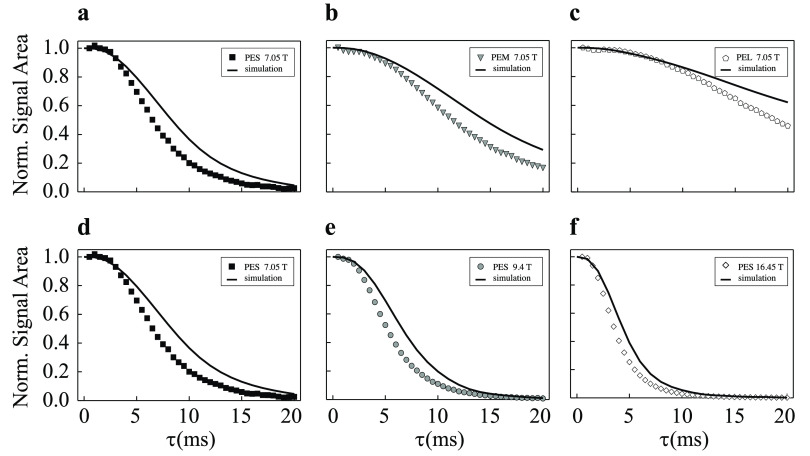
Simulated diffusion propagator calculations (solid lines) and experimental signal area (symbols) in samples (a) PES, (b) PEM, and (c) PEL at 7.05 T and for sample PES at (d) 7.05 T, (e) 9.4 T, and (f) 16.45 T (see Table 2). The signal area has been normalized so that the shortest time point has unitary area. Simulations use the parameters in Table 1 and are the result of eq 65.

However, the theory only provides the scaling behavior of the propagator, not its absolute values, since the length scale *a* is not uniquely defined. For this reason, numerical simulations have been performed on all model porous media samples in Table 2 and at each magnetic field for which experiments were available. The results of simulations done for a field of 7.05 T on the three model sample consisting of beads packing of different sizes are compared with experimental measurements in Figure 8a–c.

The agreement between simulations and experiments is quite good considering that we do not know the exact magnetic susceptibilities of the samples. The deviation from experimental measurements increases at large value of τ. This can be related to a gradually more important contribution of other relaxation mechanisms that appreciably contribute to *T*_2_ at larger τ. It is also noteworthy that the diffusive attenuation effects are less severe as the beads sizes (and therefore the pore size) is increased. This is due to the fact that for large pore size the spin have to diffuse much further before to appreciate the fluctuation nature of the demagnetizing magnetic field due to susceptibility inhomogeneities. In parts d–f of Figure 8, we have compared simulations and experiments for the model porous media sample containing the smallest beads (and therefore the smallest pore sizes) for three values of the magnetic field, namely 7.05, 9.4, and 16.45 T, from parts d to f, respectively.

Once again, we note a very good agreement between simulations and experiments which confirm the good quality of our simulation approach. As expected, the diffusive attenuation effects are more severe as the field is increased because the demagnetizing field depends linearly on the static magnetic field.

**Figure 9:**
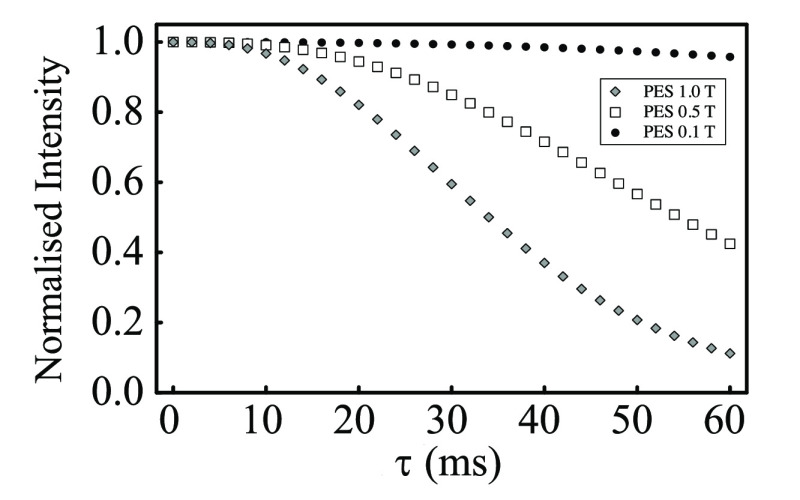
Simulated diffusion propagator calculations for sample PES at 1 T (diamonds), 0.5 T (squares), and 0.1 T (spheres). Simulations use the parameters in Table 1 and are the result of eq 65.

In order to estimate at which magnetic field these effect becomes negligible at long echo times that would be required, for example, in a M2S/S2M experiment, we have simulated the effects of diffusive attenuation for the PES sample at 3 smaller values of the static magnetic field, namely 1, 0.5, and 0.1 T. The results of these simulations are reported in Figure 9.

It becomes clear that the diffusive attenuation at echo time duration of, say 30 ms, requires the experiments to be taken at magnetic fields of 0.1 T or lower when molecules are diffusing between the pores resulting from the packing of 212–250 μm plastic spheres, for which a susceptibility mismatch of 2.71 ppm exists between the structure and the solvent. The simulation framework is therefore very useful in estimating these properties for any actual porous structure providing that the involved magnetic susceptibilities are known and an accurate image of the sample can be acquired either through CT scan, as in this work, or via MRI and indeed any other available techniques.

## Conclusions

A theoretical framework has been developed to predict the diffusive attenuation of transverse nuclear spin order in liquids imbibed into random media. Experimental measurements have been performed on densely packed polyethylene beads of varying size and at a variety of magnetic fields. The scaling of the resulting attenuation with bead size as well as with the magnetic field agrees closely with theoretical predictions derived by neglecting correlations between the susceptibility inhomogeneities and molecular diffusion.

In order to provide a quantitatively accurate prediction, a simulation framework has been developed that departs from CT images of the 3D structure of the sample. The CT data is used to compute the inhomogeneous demagnetizing field, and as a framework for Brownian dynamics simulation of molecular diffusion. This provides time-series data for the magnetic field experienced by a diffusing molecule, from which the required ensemble averages can be computed. The predictions were found to be in excellent agreement with experimental observations. The theoretical and simulation framework presented here enables systematic predictions of the diffusive attenuation behavior of more complex spin states. In turn, this is of great importance in the design of experimental strategies for magnetic resonance techniques based on long-lived spin states and their applications to study random media. As the simulation framework does *not* neglect correlations between magnetic field and diffusion, it may also be applied to low and intermediate field regimes where such effects are expected to be relevant. Actually, such experiments are currently underway in our laboratory and will be reported separately.
